# Health-related quality of life and hospital costs following esophageal resection: a prospective cohort study

**DOI:** 10.1186/s12957-015-0678-3

**Published:** 2015-09-04

**Authors:** C. Strik, R. P. ten Broek, M. van der Kolk, H. van Goor, J. J. Bonenkamp

**Affiliations:** Department of surgery, Radboud University Medical Center, postal number 618, P.O. Box 9101, 6500 HB Nijmegen, Netherlands

**Keywords:** Postoperative complications, Esophageal surgery, Cost analysis

## Abstract

**Background:**

The incidence rates for adenocarcinoma of the esophagus are increasing while the prognosis has only improved slightly. There is no apparent benefit in short- and long-term survival after different surgical strategies, but surgery is associated with significant morbidity. The goal of this study is to prospectively assess the quality of life and hospital costs after esophageal resections depending on the development of complications.

**Methods:**

Prospective data was collected from 47 patients undergoing an esophageal resection for esophageal cancer participating in the prospective LAParotomy or LAParoscopy and Adhesions (LAPAD) study (clinicaltrials.gov registration number: NCT01236625). A comparison was made between patients who developed major complications and minor or no complications regarding quality of life and hospital costs.

**Results:**

Thirteen patients developed major complications while 34 patients developed only minor or no complications. Patients with major complications had a mean hospital cost of $16,369 vs $12,409 for patients without or with minor complications. We found no difference in quality of life between the two groups 6 months after surgery.

**Conclusions:**

In our cohort, major complications did not seem to have a detrimental effect on postoperative quality of life 6 months after surgery but they increased costs associated with esophageal resection.

## Synopsis

The aim of this study is to assess the quality of life and hospital costs after esophageal resection with regard to postoperative complications.

## Background

The incidence rates for adenocarcinoma of the esophagus increase in several Western countries, in part due to increases in the prevalence of known risk factors such as overweight and obesity. An estimated 482,300 new esophageal cancer cases and 406,800 deaths occurred in 2008 worldwide [1].

The prognosis of esophageal cancer has only slightly improved [2]. Surgical resection is the only treatment with curative intent. Population-based studies from Europe show 5-year survival rates after curatively intended surgery for esophageal adenocarcinoma of 30.7 %. The population-based stage-specific 5-year survival is 67, 33, and 8 % in stages 0–I, II, and III, respectively. There is no apparent benefit in short- and long-term survival between the different surgical treatment strategies [3–7].

Especially for treatments with significant morbidity, quality of life and hospital costs are important outcome parameters. Surprisingly, accurate data on quality of life and hospital costs and their association with complications following esophageal surgery are scarce [8–10].

The aim of this study is to prospectively assess the quality of life and the costs of neo-adjuvant chemo radiotherapy followed by an open esophageal resection and the effect of a complicated postoperative course on these outcomes.

## Methods

For this study, we utilized data of consecutive patients with open transhiatal resection of esophageal carcinoma participating in the prospective LAParotomy or LAParoscopy and Adhesions (LAPAD) study (clinicaltrials.gov registration number: NCT01236625). Detailed methods of the LAPAD study are previously reported [11]. The LAPAD study included all patients admitted to the surgical ward of the Radboud University Medical Center for elective laparotomy or laparoscopy between June 2008 and June 2010. Detailed data on demographics, per- and postoperative morbidity, hospital costs, and health-related quality of life (HRQoL) of patients with curative surgery for esophageal carcinoma was extracted from the LAPAD database. Tumor histology and grade were reviewed in the pathology report.

### Patient selection

Patients with resection for esophageal carcinoma were selected using the ICD-10 code at admission (C15*, C160, or D130). For the purpose of differentiating between patient with or without major postoperative complication, the Clavien-Dindo Classification of Surgical Complications was used [12]. Complications scored as grade II or higher were considered major complications. HRQoL was only studied in patients who filled in a questionnaire at hospital admission or at 6 months follow-up.

### Health-related quality of life

HRQoL was assessed using three validated questionnaires: the short-form 36 (SF-36), the Duke Activity Status Index (DASI), and the gastro-intestinal complaints list (GIC). The structure and validity of the SF-36, DASI, and GIC have been reported previously [13–15].

HRQoL questionnaires were completed the day before surgery and at 6 months after discharge. Follow up at six months was obtained by sending a postal questionnaire with cover letter. Patients received a postal reminder after 3 weeks and a telephonic reminder after an additional 3 weeks.

### Cost analysis

Costs analysis was performed in US dollars and included only the direct hospital costs: operation costs, ward stay, ICU stay, extra charges for parental and tube feeding, postoperative diagnostic procedures, reoperation costs, and blood products. Costs calculations were performed using the guidelines for cost analysis from the Dutch College of Health Insurance Companies using a top-down approach. Operation costs were calculated based on total anesthesia time with operating room costs of $1390/h including personnel, material, and overhead costs. Total costs for the surgical ward and ICU were $661 and $2289 per day, respectively, and included basic nutrition costs. More than basic parental and tube feedings were calculated as extra nutritional costs. Diagnostic and reoperation costs were calculated using the price lists for medical procedures by the Dutch College of Health Insurance Companies of 2004. Medication costs and blood products costs were calculated according to the standardized price list by the Dutch College updated for June 2008 [16, 17].

### Sample size calculation

Sample size calculation was based on the primary outcome SF-36 score 6 months post-operatively. We defined a difference of 10 % in the postoperative SF-36 score between patients with severe complications compared to patients with minor or no complications as relevant. The population standard deviation was estimated at 10 %. Based upon previous literature, the incidence of major complications was estimated at 50 % [4]. Using these assumptions, 32 patients were required to detect a 10 % difference in SF-36 score 6 months post-operatively with 80 % power. Accounting for a 20 % loss to follow-up, a minimum of 43 patients form the LAPAD database had to be included.

### Statistical analysis

Comparison was made between patients with (Clavien-Dindo grade II or more) or without a major complication (Clavien-Dindo grade 0 and I). Dichotomous data was analyzed using *χ*^2^ test. Continuous data was presented as means and tested using an independent *T* test. If continuous data was not normally distributed, the Mann-Whitney test was used for comparison of groups. A Wilcoxon signed-rank test was used to evaluate statistical significant differences between the pre- and postoperative data within the two groups. We used the Kolmogorov-Smirnov test to assess whether data was normally distributed. For the statistical analysis of costs, we used a log transformation to correct for a non-normal distribution in order to use an independent *T* test. A *p* value < 0.05 was considered significant. Statistical analysis was performed using SPSS version 17.0 for Windows.

## Results

Eight hundred forty-four planned operations were screened for eligibility during the LAPAD study, and 750 operations were included. In 47 patients, the inclusion criterion of admission and resection of esophageal carcinoma was met. Data on postoperative morbidity and costs were available in all patients. Of 47 included patients, 7 patients did not complete all quality of life questionnaires, 1 patient did not complete the SF-36 and GIC questionnaire, and 1 patient did not complete the GIC questionnaire. Another 4 patients were not able to complete their quality of life questionnaires after 6 months. Thus, data from 34 (72 %) patients was available for pre and postoperative HRQoL assessment.

In-hospital mortality was 0 %. Thirteen patients (28 %) developed complications of Clavien-Dindo grade II or higher, and 34 (72 %) patients did not develop a complication or only a minor complication. There were no statistically significant differences between the two groups in patient (Table 1) and tumor (results not shown) characteristics. Particularly, co-morbidity did not differ between groups (*P* = 0.30, Table 1). Of 13 patients, 1 developed 2 major complications, which were heart failure and pneumonia. Pneumonia was present in 7 patients, 1 patient developed anastomotic leakage, 1 fascial dehiscence, 1 nephrogenic diabetes insipidus, and 1 postoperative hemorrhage. Two patients with major complications developed in addition a superficial wound infection classified as a minor complication. Of the 34 remaining patients (72 %), 29 had an uncomplicated postoperative course, 5 had minor complications (Clavien-Dindo grade I); 1 patient had a urinary tract infection, 2 needed additional diuretics because of cardiac problems, and 2 developed a postoperative ileus.

**Table 1:** Patient characteristics and costs

Patient characteristics	Total number of patients	Patients with major complications	Patients without major complications	
Number of patients	47 (100 %)	13 (28 %)	34 (72 %)	
Age	64.02	61.77	64.88	*P* = 0.27
Sex (M/F)	41/6	12/1	29/5	*P* = 0.66
BMI	26.09	27.10	25.71	*P* = 0.15
P-POSSUM	8.40	8.24	8.47	*P* = 0.95
Co-morbidity	37 (78.7 %)	11 (84.6 %)	26 (76.5 %)	*P* = 0.30
Complications				
Pneumonia	8	8	0	
Anastomotic leakage	1	1	0	
Postoperative hemorrhage	1	1	0	
Platzbauch	1	1	0	
Respiratory failure	1	1	0	
Decompensatio cordis	1	1	0	
Minor complications	7	2	5	
Outcome				
Re-interventions	3	3	0	
In-hospital mortality	0	0	0	
Surgical ward stay	9.12	11.30	8.29	*P* = 0.06
Medium-care stay	0.76	1.06	0.64	*P* = 0.41
IC-stay	1.52	1.78	1.41	*P* = 0.36
Total hospital stay	11.39	14.16	10.33	*P* = 0.04
TPV-requirement	1 (2.1 %)	1 (7.7 %)	0 (0 %)	*P* = 0.28
Number of readmittances to hospital	5 (10.6 %)	0 (0 %)	5 (14.4 %)	*P* = 0.30
Costs				
Operation		4062	4042	*P* = 0.73
Surgical ward		5379	3944	*P* = 0.15
Medication		638	319	*P* = <0.01
Diagnostic		433	251	*P* = 0.04
Microbiology		119	27	*P* = 0.04
Total cost		16,369	12,409	*P* = 0.02

The group with major complications had a mean total days in hospital of 14.2 days compared to 10.3 days in the group without or with minor complications (*P* = 0.04; Table 1).

**Fig. 1:**
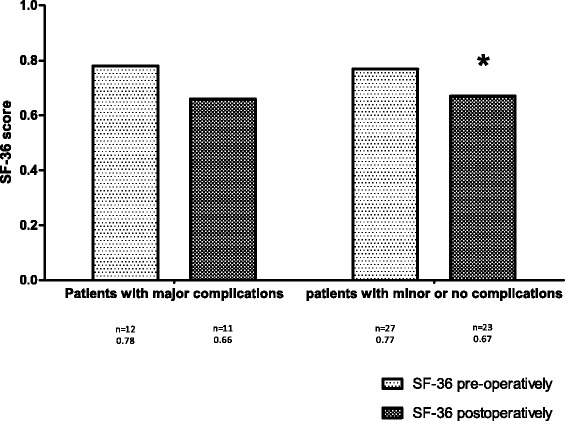
Results of the pre- and postoperative SF-36 questionnaire

**Fig. 2:**
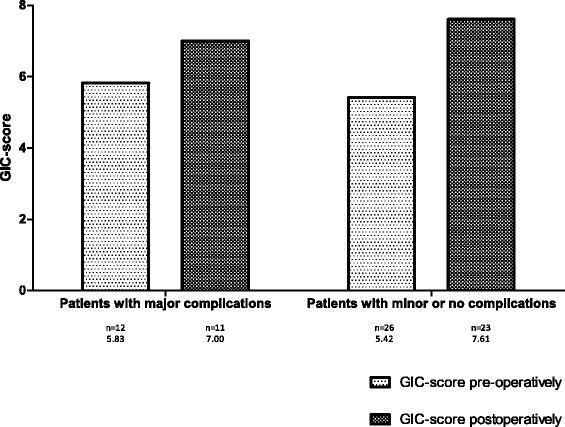
Results of the pre- and postoperative GIC questionnaire

**Fig. 3:**
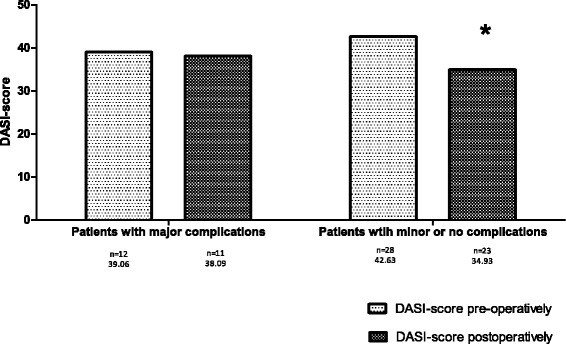
Results of the pre- and postoperative DASI questionnaire

There was a non-significant decline in QoL 6 months post-operatively for all three questionnaires in the group with major complications (Figs. 1–3). The decline in QoL in the group without or with minor complications was significant for the SF-36 and DASI (0.8 and 42.6 compared to 0.7 and 34.9; Figs. 2 and 3). The scores of the QoL questionnaires did not show any statistically significant differences between the two groups. No statistical differences between the different domain scores of the SF-36 questionnaire and the different component scores of the GIC questionnaire were found, pre- and 6 months post-operatively, between both groups (results not shown). The group with major complications did not show a greater decline in QoL at 6 months post-operatively in comparison to the group without or with minor complications.

Costs are presented in Table 1. Total costs reached a significant difference; $12,409 for the group without or with minor complications vs $16,369 for the group with major complications (*p* = 0.02). This was predominantly based on higher costs for microbiology, $27 vs $119 (*p* = 0.04), costs associated with medication, $319 vs $638 (*p* = <0.01), and diagnostic costs, $251 vs $433 (*p* = 0.04). There was a trend towards higher costs for the surgical ward (*p* = 0.15).

## Discussion

This is the first study to our knowledge that prospectively investigated the relation between postoperative complications and quality of life. It demonstrates that health-related quality of life declines after esophageal resection for carcinoma but irrespectively of the development of early postoperative major complications. We showed higher costs and a longer hospital stay associated with major complications.

Our study showed a 0 % in-hospital mortality, a 17 % incidence of pneumonia, and a low overall incidence of complications compared to the literature. A recent randomized controlled found a 12 % incidence of pneumonia for patients undergoing minimal invasive esophageal resection [18] while open (transhiatal or transthoracic) esophageal resection is associated with a 27 and 57 % incidence of pulmonary complications [4]. The low complication rate in our institute might be explained by a dedicated multi-disciplinary team approach. The pre-operative work-up strategy in our center consists of pre-operative dietary measures and physiotherapy combined with admittance to the ICU for goal directed hemodynamic and respiratory optimization the day before surgery. Complications and in-hospital mortality rates remained comparably low in the years after study closure.

The development of postoperative complications did not result in a significant decrease in QoL in this study. This conclusion should be interpreted with some caution as the low incidence of major complications resulted in a smaller group of patients that developed major complications than accounted for in our sample size calculation. However, we did not find a statistical significant difference between the groups; on the contrary, the population estimates of SF-36, gastro-intestinal complaints score, and DASI-score were highly comparable between the groups. The most common complication was a pneumonia, and although this is a major complication with a negative impact on 5-year survival [19], patients recover completely when treated adequately [20]. It is possible that complications requiring re-intervention and or admission to the intensive care unit have a more detrimental effect on QoL, but our study could not demonstrate this effect because of the low incidence of these complications and a high standard deviation in the SF-36 score.

We showed that there is a statistically significant difference in total hospital costs for patients who developed major complications. Kuppusamy et al. also showed a difference in costs between patients who developed complications and who did not develop complications undergoing esophageal resection [21]. However, the study population consisted of four small groups of 15 patients undergoing different treatment regimens. Kuppusamy et al. used a financial cost-accounting system that also encompassed indirect costs. These indirect costs consist of a complex association of all overhead costs, including billing, information systems, finance, and administration. These costs are hospital specific and not applicable in general. Although actual costs may vary per country, our cost calculation utilized only the direct true hospital costs, making our results universally applicable. Our study found in particular a difference in diagnostic and medication costs. This is because most patients with a major complication developed a pneumonia treated with antibiotics.

A limitation of this study is that it did not use the European Organization for Research and Treatment of Cancer Quality of Life Questionnaire (EORTC QLQ)-OES18 because the LAPAD study included all types of laparotomies. However, the gastro-intestinal complaints questionnaire has six items that are similar to the EORTC QLQ-OES18, and it is a validated QoL questionnaire. Although our study population is small, it is a consecutive group of patients with characteristics encountered in all hospitals that perform esophagectomies. Therefore, we believe that our results apply to all patients undergoing an esophageal resection for cancer in hospitals with a similar quality of care.

## Conclusions

In our cohort, major complications did not seem to have a detrimental effect on postoperative quality of life 6 months after surgery but they increased costs associated with esophageal resection.

## Abbreviations

DASI: Duke Activity Status Index
EORTC QLQ: European Organization for Research and Treatment of Cancer Quality of Life Questionnaire
GE-junction: gastro-esophageal junction
GIC: gastro-intestinal complaints list
HRQoL: health-related quality of life
LAPAD: LAParotomy or LAParoscopy and Adhesions study
SF-36: short-form 36
